# The Unconserved Groucho Central Region Is Essential for Viability and Modulates Target Gene Specificity

**DOI:** 10.1371/journal.pone.0030610

**Published:** 2012-02-03

**Authors:** Wiam Turki-Judeh, Albert J. Courey

**Affiliations:** Department of Chemistry and Biochemistry and Molecular Biology Institute, University of California Los Angeles, Los Angeles, California, United States of America; University of Florida, United States of America

## Abstract

Groucho (Gro) is a *Drosophila* corepressor required by numerous DNA-binding repressors, many of which are distributed in gradients and provide positional information during development. Gro contains well-conserved domains at its N- and C-termini, and a poorly conserved central region that includes the GP, CcN, and SP domains. All lethal point mutations in *gro* map to the conserved regions, leading to speculation that the unconserved central domains are dispensable. However, our sequence analysis suggests that the central domains are disordered leading us to suspect that the lack of lethal mutations in this region reflects a lack of order rather than an absence of essential functions. In support of this conclusion, genomic rescue experiments with Gro deletion variants demonstrate that the GP and CcN domains are required for viability. Misexpression assays using these same deletion variants show that the SP domain prevents unrestrained and promiscuous repression by Gro, while the GP and CcN domains are indispensable for repression. Deletion of the GP domain leads to loss of nuclear import, while deletion of the CcN domain leads to complete loss of repression. Changes in Gro activity levels reset the threshold concentrations at which graded repressors silence target gene expression. We conclude that co-regulators such as Gro are not simply permissive components of the repression machinery, but cooperate with graded DNA-binding factors in setting borders of gene expression. We suspect that disorder in the Gro central domains may provide the flexibility that allows this region to mediate multiple interactions required for repression.

## Introduction

As corepressors, *Drosophila* Groucho (Gro) and its orthologs in other metazoans lack DNA-binding domains and are recruited to the template by numerous DNA-binding repressor proteins including HES family repressors, Engrailed, Dorsal, Capicua (Cic), and Brinker (Brk) [1], [2], [3], [4], [5]. Once recruited to the template, Gro regulates a variety of developmental processes including neurogenesis, sex determination, and patterning of the embryo and imaginal discs [6]. In addition, Gro regulates signaling through multiple signal transduction pathways, including the Ras, Notch, Wingless, and Decapentaplegic (Dpp) pathways [7]. Mammalian Gro orthologs exhibit similarly widespread roles in signaling and development and have been implicated in tumorigenesis [8], [9].

Many of the DNA binding repressors through which Gro acts, including Dorsal, Cic, and Brk, are distributed in concentration gradients that provide positional information along developmental axes. A graded factor is able to subdivide fields of developing cells because different target promoters respond to different threshold concentrations of the factor and are therefore expressed in domains with differing borders. Previous efforts to understand how target promoters can respond to different concentrations of DNA binding transcription factors have usually focused on the role of the DNA binding factors themselves, with particular attention to such parameters as binding site affinity, cooperative binding to DNA, synergy and antagonism between DNA bound factors, etc. [10], [11], [12], [13]. Broadly distributed co-regulators, such as Gro, have generally been viewed as required components of the regulatory system that are needed for activation or repression by graded DNA binding transcription factors, but that do not have active roles in target gene selection or in determining borders of target gene expression.

Gro/TLE family members are characterized by a conserved N-terminal domain (the Q domain), a variable middle region that can be subdivided into GP, CcN, and SP domains, and a conserved C-terminal WD-repeat domain [14]. Sequencing of lethal Gro point mutant alleles has revealed mutations that map to the WD repeat and Q domains, demonstrating the functional importance of these two domains [15], [16]. In contrast, none of the known point mutations map to the GP, CcN, or SP domains, suggesting that these regions may not be required for viability [16]. However, an alternative possibility, which is suggested by the poor conservation in these regions, is that they are not well ordered and are therefore resistant to inactivation by point mutagenesis.

The roles of the conserved Q and WD-repeat domains are well studied. The Q domain is required for Gro homo-oligomerization, and point mutations within this domain that disrupt self-association also interfere with Gro-mediated repression [17], [18]. This region also binds to several repressors including Tcf/Lef and Myc [19], [20]. The WD-repeat domain forms a β-propeller that contacts peptide motifs found in many Gro-binding corepressors, and thus is critical for the recruitment of Gro to many of its target genes [2], [4], [15], [21].

Less is known about the poorly conserved central region. This region, along with the Q domain, mediates binding of Gro to hypoacetylated histone N-terminal tails [22]. The GP domain binds the histone deacetylase Rpd3/HDAC1, which is required for optimal Gro function [23], [24], [25]. The CcN domain contains a putative nuclear localization signal (NLS), as well as phosphorylation sites for the cyclin-dependent kinase family member Cdc2 and casein kinase II (CKII) [14]. Association of Gro with chromatin is negatively regulated by Cdc2 phosphorylation and positively regulated by CKII phosphorylation of the CcN domain [26], [27]. The SP domain contains phosphoacceptor sites for mitogen activated protein kinase (MAPK) [28], [29] and homeodomain-interacting protein kinase (HIPK) [30], [31]. Phosphorylation of Gro by both these kinases leads to decreased transcriptional repression, although the mechanism behind this phenomenon is unclear.

A model for Gro-mediated repression that incorporates some of the above-described biochemical functions of its domains is as follows: Once Gro is recruited via interactions with repressors, its ability to self-associate and to bind histone tails allows it to polymerize along the template establishing a transcriptionally silent domain [32]. The template bound Gro may then recruit histone deacetylase Rpd3 leading to histone deacetylation and possibly to an increase in nucleosome density [25], [33]. In support of this model, Gro family proteins are able to condense chromatin arrays in vitro thereby preventing the transcriptional machinery from having access to the template [34]. Any or all of these steps could be regulated by phosphorylation of the CcN and SP domains. While the above pathway may account for many examples of Gro-mediated repression, additional findings suggest that Gro may repress by other mechanisms as well, including histone deacetylase independent mechanisms [25], [34], [35], [36]. Further evidence for mechanistic diversity comes from studies showing that Gro may function in both long- and short-range repression [37], [38]. It appears that mechanisms of Gro-mediated repression may vary depending on the target gene and developmental context.

In this study, we examined the roles of the unconserved Gro central domains in Gro-mediated repression and pattern formation by analyzing multiple Gro deletion variants lacking one or more of the central domains. Our findings indicate that the SP region has a negative role in repression and helps to determine target gene specificity, while the GP and CcN domains are required for repression and viability, thus challenging the notion that essential function and evolutionary conservation go hand in hand. Our studies also indicate that development requires the correct balance between positively and negatively acting Gro domains as excess Gro activity results in the inappropriate resetting of repressor concentration thresholds sufficient to mediate repression. This implies that ubiquitous co-regulators such as Gro may actively cooperate with graded DNA-binding transcription factors in setting the boundaries of developmental domains. Our findings further suggest that such factors cannot be neglected in efforts to predict the targets of DNA binding transcription factors and to predict how such targets will respond to developmental cues.

## Results

### The Gro central domains are likely to be intrinsically disordered

**Figure 1:**
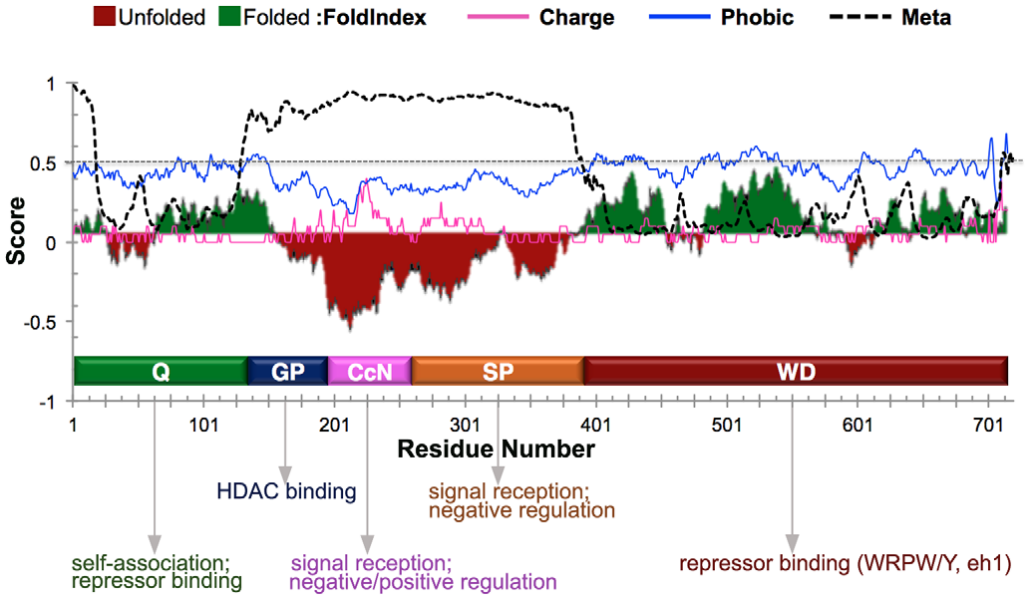
The central domains mediate multiple essential functions and are likely disordered. Prediction of disorder in Gro using two disorder prediction algorithms: PONDR-FIT™ (Meta, black dashed line) [40] and FoldIndex© (green and red shaded plot) [41]. FoldIndex© tool also calculated hydrophobicity (blue line) and charge density (pink line). Residues with Meta scores exceeding 0.5 are likely to be disordered, as are residues with FoldIndex© scores below 0. The prediction tools strongly suggest that the Gro central region is disordered, while the Q and WD-repeat domains are ordered. The Gro domains (shown along the horizontal axis) are labeled with the many of the functions that have been previously ascribed to them (see text for references).

The lack of conservation in the Gro central region suggests that this region might not be highly ordered. To explore this idea, we analyzed the Gro amino acid sequence using well-established algorithms for predicting protein disorder [39], [40]. This analysis shows that the central domains display all the hallmarks of intrinsically disordered protein domains – in particular they contain a high proportion of charged amino acid residues and a low proportion of hydrophobic amino acid residues [40], [41] (Figure 1). Numerous predictors of disorder indicate that, while the Q and WD repeat domains are ordered, the GP, CcN, and SP domains are all very likely to be disordered (Figure 1).

### Gro central domains are not required for repressor binding or self-association

**Figure 2:**
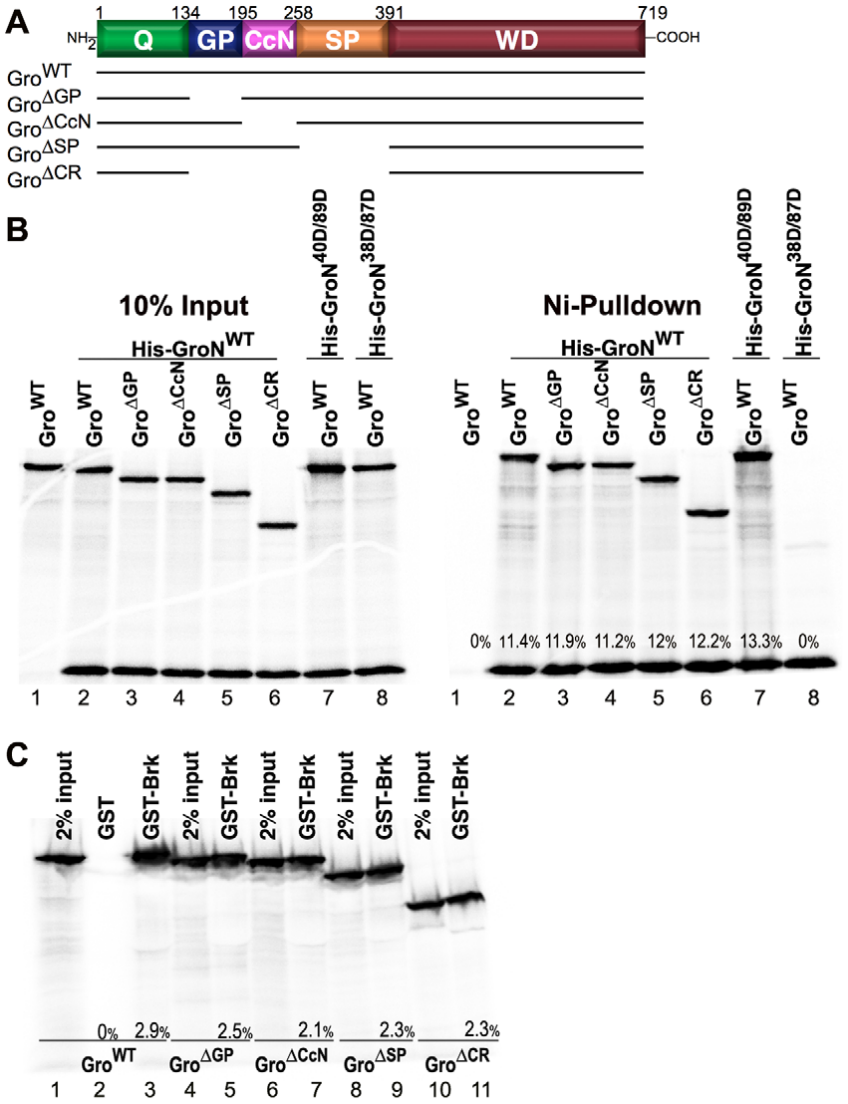
Central domain deletions impair neither Q nor WD-repeat domain function. (A) Structure of the Gro deletion variants, Gro^ΔGP^, Gro^ΔCcN^, Gro^ΔSP^, and Gro^ΔCR^. (B) Gro self-association assays. Untagged wild-type full-length Gro (lanes 1, 2, 7 and 8) or internal deletion variants of Gro (lanes 3–6) were translated alone (lane 1); or cotranslated with the wild-type His-tagged Gro N-terminal region (His-GroN^WT^, contains the first 194 amino acids of Gro including the intact Q domain) (lanes 2–6), with the 40D/89D double point mutant form of the His-tagged Gro N-terminal region (His-GroN^40D/89D^) (Lane 7), or the 38D/87D double point mutant form of the His-tagged Gro N-terminal region (His-GroN^38D/87D^) (Lane 8). Samples were incubated with Ni-NTA beads, and after extensive washing, Ni-bound proteins were eluted and analyzed by 10% SDS-PAGE and autoradiography. 10% of input is shown on the left, while the Ni-bound proteins are shown on the right. The percentage of input that was immobilized on the beads is indicated at the bottom of each lane. (C) GST-Brk pulldown assays using Gro deletion variants. [^35^S]-methionine-labeled Gro^WT^ (lanes 1–3), Gro^ΔGP^ (lanes 4–5), Gro^ΔCcN^ (lanes 6–7), Gro^ΔSP^ (lanes 8–9), or Gro^ΔCR^ (lanes 10–11) were incubated with glutathione-agarose beads bearing immobilized GST (lane 2) or a GST-Brk fusion protein (lanes 3, 5, 7, 9 and 11). After extensive washing, bound proteins were eluted and analyzed by 8% SDS-PAGE and autoradiography. The percentage of input protein that bound the glutathione-agarose beads is indicated at the bottom of the GST and GST-Brk lanes.

To test the idea that the lack of lethal point mutations in the central domains reflects disorder (and therefore an ability to tolerate single amino acid changes) rather than a lack of function, we set out to examine both the recessive and dominant phenotypes associated with transgenes encoding Gro deletion variants lacking one or more of the central domains (Figure 2A). Since the aim of these experiments was to identify essential functions for the Gro central domains, it was necessary to show that the functions of the conserved Q domain in self-association and the conserved WD-repeat domain in repressor binding were not adversely impacted by the internal deletions. Accordingly, before examining the effects of these deletions in vivo, we expressed the deletion variants in vitro and assessed their ability to self-associate and to bind a repressor protein (Brk).

In the self-association assays (Figure 2B), full-length Gro or internal deletion variants of Gro were cotranslated with an N-terminal 194 amino acid residue long His-tagged Gro fragment containing the intact Q domain (His-GroN^WT^). The His-tagged protein was immobilized on Ni-NTA beads and co-immobilization of the ^35^S-labeled untagged internal deletion variants was assessed by SDS-PAGE followed by autoradiography. Immobilization of the untagged variants was completely dependent on the presence of His-GroN^WT^ (compare lanes 1 and 2), while full length Gro and all the internal deletion variants bound His-GroN^WT^ with comparable efficiency (lanes 2–6). The specificity of the assay was demonstrated by the results obtained with His-GroN^40/89D^ and His-GroN^38/87D^. Both of these double mutants contain amino acid substitutions in the Q domain coiled-coil motifs required for self-association. In accord with previous findings, the mutations in His-GroN^40/89D^ do not inhibit self-association (lane 7), while the mutations in His-GroN^38/87D^ do (lane 8) [18].

In the GST-Brk pull down assays (Figure 2C), in vitro translated full-length Gro or internal deletion variants of Gro were incubated with a GST-Brk fusion protein immobilized on glutathione beads. Full-length Gro and all internal deletion variants bound GST-Brk with comparable efficiency (lanes 1 and 3–11). The specificity of the assay is demonstrated by the failure of full-length Gro to bind to GST alone (lane 2).

Thus, we conclude that deletion of the central domains does not interfere with the essential functions of the conserved Q and WD-repeat domains in self-association and repressor binding. Therefore, any effects of these deletions on repression most likely reflect roles of the internal domains in other biochemical interactions essential for repression.

### The Gro central domains are required for viability

**Figure 3:**
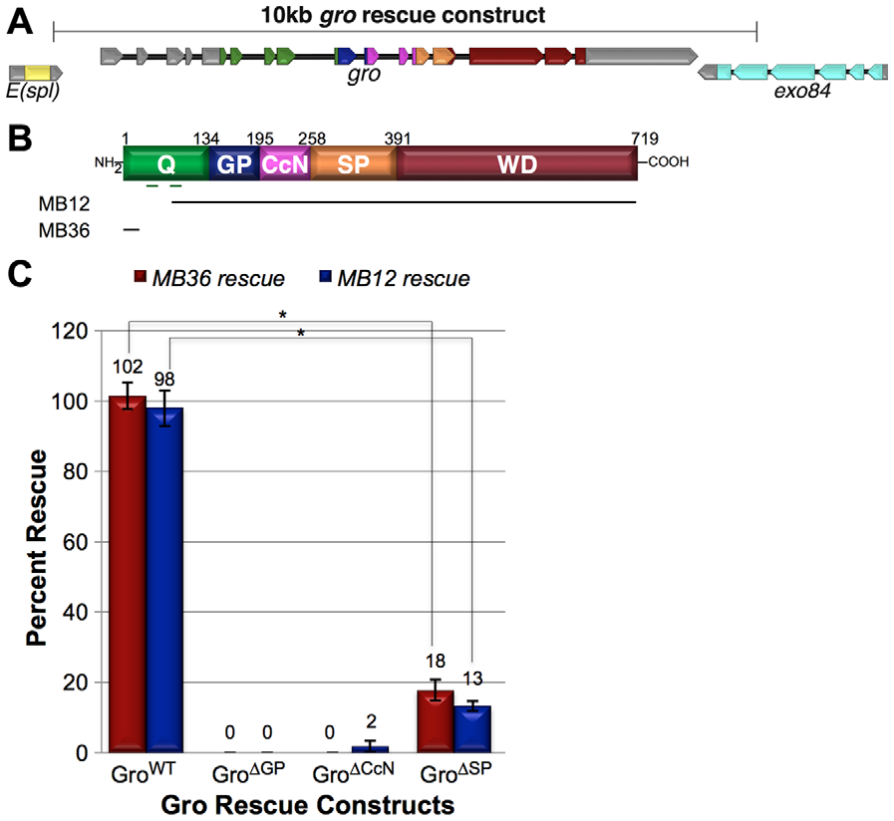
Rescue of gro mutant alleles with genomic rescue constructs encoding Gro deletion variants. (A) Intron/exon organization of *gro* and flanking regions from the left arm of chromosome 3. The 10 kb region used in the rescue constructs is indicated by the bracket. Exons encoding to the Q domain are in green, GP domain in blue, CcN domain in magenta, SP domain in orange, WD repeat domain in red, and non-encoding exons in gray. (B) The hypomorphic (*MB12*) and null (*MB36*) *gro* alleles [16]. The black lines indicate the sequences included in each deletion variant or mutant allele. The green lines represent the coiled-coil motifs in the Q domain that are known to be required for self-association [18]. (C) The indicated Gro rescue constructs were tested, as described in Materials and Methods, for their ability to rescue the lethality associated with the MB12 or MB36 *gro* alleles. Each data point is the average (± S.D.) of three independent trials (for each of trials 1 and 2, 120 flies were analyzed; for trial 3, 100 flies were analyzed). Asterisks (*) signify p<10^−5^ as determined from the two-tailed unpaired student's T test.

To determine if the central domains are required for viability, we generated genomic constructs encoding full-length Gro (Gro^WT^), or Gro deletion variants lacking the GP, CcN, or SP domains (Gro^ΔGP^, Gro^ΔCcN^, and Gro^ΔSP^, respectively) in a vector containing the bacterial attachment site (attB) for bacteriophage phiC31. These were then introduced into the fly germ line by site-specific integration into a second chromosome phiC31 attachment site (attP) [42]. The genomic construct encoding Gro^WT^ is approximately 10 kb in length and includes the *gro* transcription unit as well as flanking sequences reaching to the neighboring genes both upstream and downstream of the *gro* transcription unit (Figure 3A). In the course of introducing the full-length genomic construct into flies, we noticed that the flies were surprisingly sensitive to *gro* gene dosage. Specifically, we found that, while flies could tolerate a single copy of the *gro* transgene (in a background containing two wild-type copies of endogenous *gro*), a second copy of the transgene resulted in complete lethality.

To assess the recessive phenotypes of the deletion variants, the transgenes encoding Gro^WT^, Gro^ΔGP^, Gro^ΔCcN^, and Gro^ΔSP^ were crossed into flies homozygous for either of two lethal alleles of *gro* (the null *gro^MB36^* allele or the hypomorphic *gro^MB12^* allele [16], Figure 3B). The construct encoding Gro^WT^ completely rescued the lethality associated with either *gro* allele (Figure 3C). In contrast, constructs encoding Gro^ΔGP^ and Gro^ΔCcN^ were unable to rescue the lethal *gro* alleles (Figure 3C), strongly suggesting essential roles for the GP and CcN domains in Gro function. In the presence of endogenous wild-type Gro, the Gro^ΔCcN^ rescue construct rendered the flies weak and led to wing blistering and abnormal abdominal segmentation, while Gro^ΔGP^ had no such dominant phenotype (data not shown). Although the construct encoding Gro^ΔSP^ gave some rescue, this rescue was ∼5 to 8-fold less efficient than rescue by the wild-type transgene (Figure 3C). We did not attempt to generate a rescue construct encoding Gro^ΔCR^ because preliminary misexpression experiments (see the following section) strongly suggested that this construct would lead to dominant lethality.

In conclusion, the central domains appear to have essential roles in Gro function strongly suggesting that their apparent immutability is a reflection of their disorder. To explore the functions of the central domains further, we have examined the effects of misexpression of the deletion variants in two different developmental contexts: the embryo and the wing disc.

### The GP, SP, and CcN domains are required for embryonic patterning

**Figure 4:**
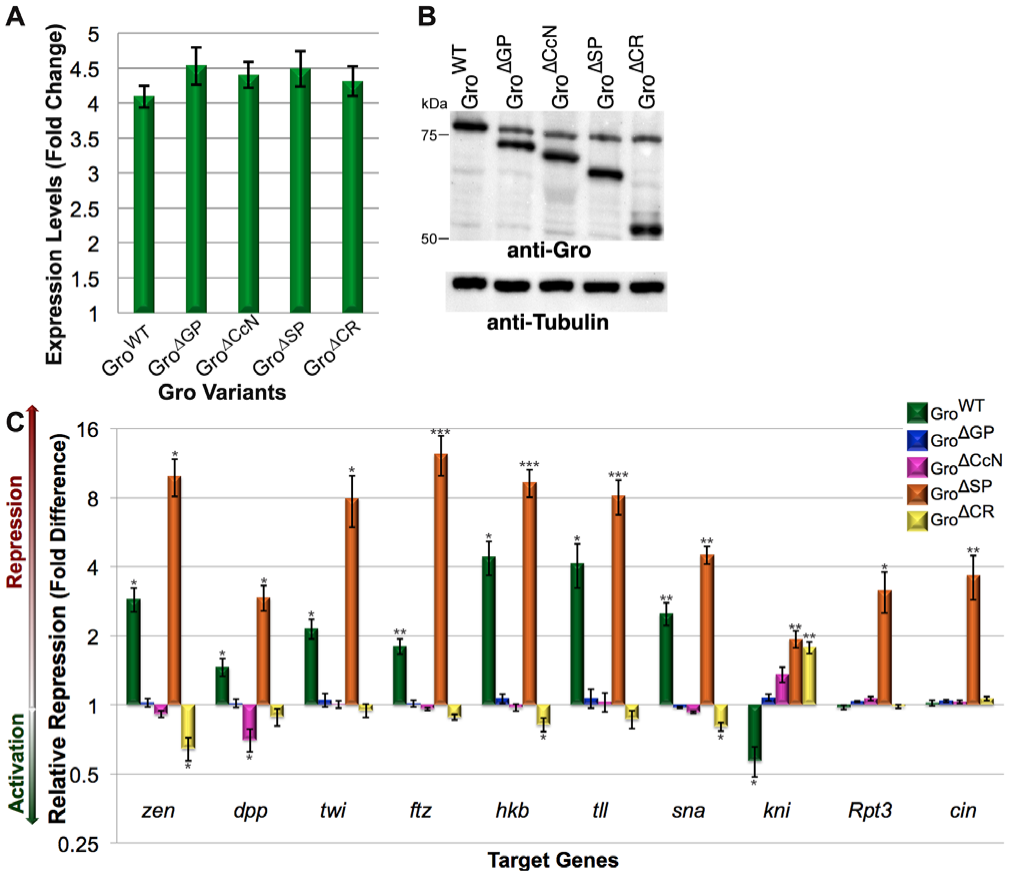
Central domains have both positive and negative roles during Gro-mediated repression in the embryo. (A) qRT-PCR analysis of the *gro* transcript in embryos shows that *Mat-Gal4* driven expression results in very similar levels of overexpression of each variant. (B) An anti-Gro immunoblot verifies equal expression of the variants. An anti-tubulin immunoblot serves as a control for relative total protein levels. (C) qRT-PCR was performed on embryos lacking maternally overexpressed Gro; or containing maternally overexpressed Gro^WT^ (green), Gro^ΔGP^ (blue), Gro^ΔCcN^ (pink), Gro^ΔSP^ (orange), or Gro^ΔCR^ (yellow). Expression levels of *zen*, *dpp*, *twi*, *ftz*, *hkb*, *tll*, *sna*, *kni*, *Rpt3*, and *cin* were normalized for expression levels of *RpL32*. Each fold repression value was obtained by dividing the normalized expression level of a gene in the absence of overexpressed Gro by the normalized expression level of the same gene in the presence of one of the overexpressed Gro variants. Values were graphed on a log_2_ scale. Statistical significance of each value relative to the value in embryos lacking overexpressed Gro was determined from the two-tailed unpaired Student's T-test. * signifies p<0.05, ** signifies p<0.01, *** signifies p<0.005. No asterisk signifies p>0.05.

To determine the roles of the central domains in embryonic patterning, we used a maternally active Gal4 driver to direct expression of UASp constructs encoding Gro^WT^, Gro^ΔGP^, Gro^ΔCcN^, Gro^ΔSP^, and Gro^ΔCR^. qRT-PCR analysis of the transcripts in 0–3 hour embryos encoding the maternally expressed Gro variants indicates that all five variants are overexpressed by ∼4-fold relative to endogenous Gro (Figure 4A), a finding that is consistent with the results of an anti-Gro immunoblot (Figure 4B).

We examined the effect of maternal overexpression of the deletion variants on the expression of a variety of early embryonic Gro targets by qRT-PCR. If expression levels of Gro target genes were sensitive to Gro levels (as opposed to just its absence or presence), overexpression of active Gro variants would be expected to reduce expression of Gro targets to levels below those seen in wild-type embryos. In accord with this expectation, overexpression of Gro^WT^ leads to repression of the Dorsal targets *zerknült* (*zen*), *twist* (*twi*), *dpp*, and *snail* (*sna*), the Hairy target *fushi tarazu* (*ftz*), and the Cic targets *huckebein* (*hkb*) and *tailless* (*tll*), and apparent activation of the Tll target *knirps* (*kni*) (Figure 4C, repression corresponds to relative repression values greater than 1, while activation corresponds to relative repression values less than 1).

The qRT-PCR assays indicate that the GP domain is critical for Gro-mediated repression, since, consistent with the genomic rescue experiments, Gro^ΔGP^ overexpression has essentially no effect on Gro target gene expression. The CcN domain is also critical for repression as Gro^ΔCcN^ overexpression leads to no repression of Gro targets and, in at least one case (*dpp*), results in significant activation of a Gro target. Similarly, overexpression of Gro^ΔCR^ (in which the entire central region is deleted) leads to no repression of Gro targets and, in several cases (e.g., *zen*, *hkb*, and *sna*), leads to significant activation of these targets. Thus, Gro^ΔCcN^ and Gro^ΔCR^ may function as dominant negatives, presumably through the formation of mixed oligomers with endogenous wild-type Gro.

Overexpression of the Gro^ΔSP^ variant resulted in significantly higher levels of repression than overexpression of Gro^WT^, indicating that the SP domain has a negative regulatory function, and explaining the incomplete rescue by Gro^ΔSP^. The relative importance of the SP domain appears to be target dependent. For example, deletion of the SP domain increases repression of Cic targets *hkb* and *tll* by a factor of ∼2, while deletion of the SP domain increases repression of the Hairy target *ftz* by a factor of more than 6.

*kni* functions as a control to show that repression due to Gro overexpression is specific for Gro targets. *kni* is not a direct Gro target, but rather it is a target for repression by Tll. Therefore, overexpression of Gro indirectly activates *kni* by directly repressing *tll*. The effects of the other deletion variants on *kni* expression are consistent with the effects of these variants on *tll* expression with the exception of Gro^ΔSP^, which unexpectedly acts to repress both *tll* and *kni*. One possible explanation for this paradox is that SP domain deletion leads to a loss of Gro specificity, turning Gro into a promiscuous repressor of genes that it does not normally repress. To explore this possibility further, we looked at two additional genes not expected to be Gro targets. Genome-wide chromatin immunoprecipitation analysis in embryos (manuscript in preparation) indicates that Gro is absent from the *Rpt3* and *cinnamon* (*cin*) loci suggesting that these genes are not likely to be Gro repression targets. As predicted, overexpression of Gro^WT^, Gro^ΔGP^, Gro^ΔCcN^, and Gro^ΔCR^ results in no change in *Rpt3* and *cin* transcript levels. In contrast, overexpression of Gro^ΔSP^ results in ∼3-fold repression of both these genes (Figure 4C). In conclusion, the SP domain seems to have a broad role in Gro specificity. In its absence, we observe increased levels of repression and promiscuous repression of genes that are not normally targeted by Gro.

**Figure 5:**
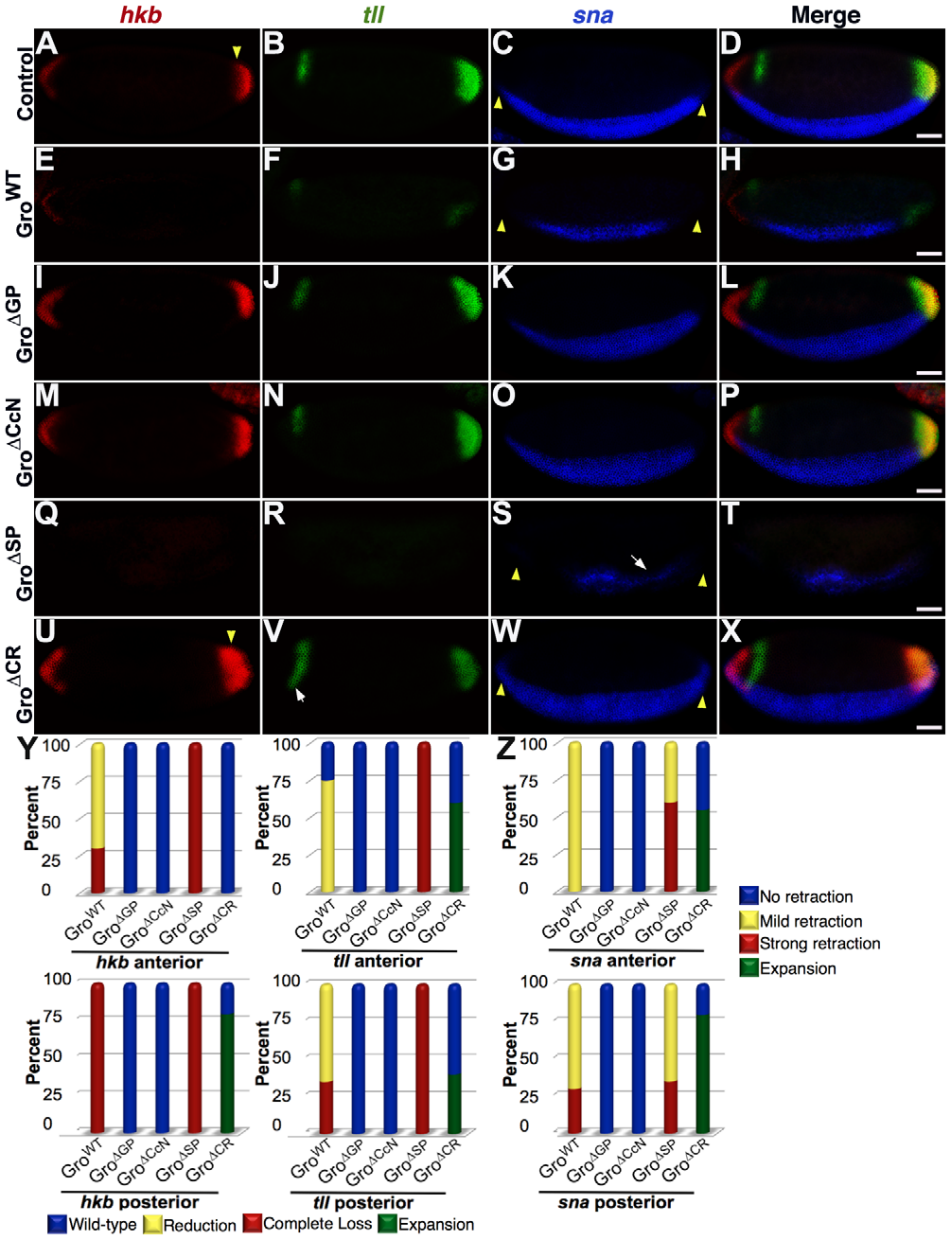
Central domains define patterns of embryonic Gro target gene expression. Fluorescence in situ hybridization analyzing the mRNA products of the Gro targets *hkb*, *tll*, and *sna* in cellular blastoderm embryos containing maternally overexpressed Gro variants. Embryos are oriented with the anterior to the left and ventral at the bottom. Embryo images were obtained using a 20× objective. Scale bars = 50 µm. (A–D) Wild-type expression patterns. (E–H) Gro^WT^ overexpression leads to spatial patterning defects and reduced expression of all three target genes. Posterior *hkb* expression was absent and anterior *hkb* expression was significantly reduced (E). Posterior and anterior *tll* expression were reduced with a greater effect at the posterior (F). The ventral *sna* stripe was narrowed ventrally and retracted from both termini (G). (I–P) Overexpression of Gro^ΔGP^ (I–L) and Gro^ΔCcN^ (M–P) led to no changes in target gene expression patterns. (Q–T) Gro^ΔSP^ overexpression led to defects in spatial patterning and a reduction in the expression of all three target genes that is more severe than that resulting from Gro^WT^ overexpression. *hkb* (Q) and *tll* (R) expression were completely abolished at both termini. The *sna* stripe was substantially narrowed in the posterior region (as indicated by the arrow) and retracted from the termini, especially the anterior terminus (S). (U–X) Gro^ΔCR^ overexpression resulted in expansion of the posterior *hkb* domain (U), and an expansion of the anterior *tll* domain toward the ventral midline of the embryo (arrow) (V). The *sna* domain is expanded towards both termini (W). Arrowheads (panels A, C, G, S, U, and W) indicate borders of the wild-type expression domains. (Y–Z) Quantification of changes in expression pattern. (Y) 20 stage 5 embryos overexpressing each variant were scored according to whether the anterior and posterior expression domains of *hkb* and *tll* appeared wild-type, were reduced, were missing altogether, or were expanded. (Z) The same 20 embryos were scored according to whether the *sna* expression domain showed no retraction, mild retraction, strong retraction, or expansion at the anterior and posterior termini.

To determine if the central region modulates the spatial patterning of embryonic target gene expression or just the overall level of expression, we performed fluorescence in situ hybridization looking at the expression of Gro targets *hkb*, *tll*, and *sna* in the cellular blastoderm embryo. Gro participates in terminal patterning by restricting the expression of *hkb* and *tll* to the embryonic termini through interaction with the repressor Cic [43], [44] (Figure 5A–B), and mediates dorsoventral (d/v) pattern formation through interaction with Dorsal, a transcription factor that can function as both an activator of genes such as *twi* and *sna* and a repressor of genes such as *dpp* and *zen* [3], [45]. Activation targets such as *sna* are expressed in a stripe around the ventral midline (Figure 5C), while repression targets such as *dpp* are expressed in the dorsal region of the embryo. The dual functionality of Dorsal relies on the fact that Dorsal has an intrinsically low affinity for Gro [46], and previous studies have shown that addition of a high affinity Gro interaction motif to Dorsal converts it into a dedicated repressor leading to *sna* repression rather than activation [47]. Targets of the d/v system, such as *sna*, are also under the regulation of the terminal pattern forming system, which works through Cic to repress these genes at the embryonic termini [43], [48]. Thus, the effects of Gro variant overexpression on *sna* expression that we describe below reflect the Gro dependence of both the terminal and dorsoventral systems.

In the following description of the effects of Gro variant overexpression on target gene expression pattern, we describe the most common phenotypes and present representative data (Figure 5A–X). We have quantified the data by looking at multiple embryos and assigning them to classes based on expression pattern (Figure 5Y–Z). The results of this quanitification are consistent with the conclusions presented below.

In the case of each target gene, overexpression of Gro^WT^ not only results in decreased levels of expression, but also leads to a disruption of transcriptional patterning consistent with the idea that excess Gro activity leads to a resetting of the threshold concentrations at which graded repressors such as Dorsal and Cic repress transcription (Figure 5E–H, compare to Figure 5A–D). *hkb* is completely repressed at the posterior terminus of embryos containing excess Gro^WT^, but only partially repressed at the anterior terminus of such embryos (Figure 5E, Y). *tll* is significantly reduced and restricted to the ventral side of such embryos at the posterior terminus and reduced at the anterior terminus (Figure 5F, Y). *sna* expression retracts significantly from the termini, and its expression domain is narrowed along the dorsoventral axis (Figure 5G, Z). In contrast, overexpression of Gro^ΔGP^ or Gro^ΔCcN^ leads to no observable changes in spatial patterning of target genes (Figure 5I–P, Y, Z) in support of the conclusion that the GP and CcN domains are essential for Gro activity.

As with overexpression of Gro^WT^, overexpression of Gro^ΔSP^ disrupts the expression patterns of *hkb*, *tll* and *sna* (Figure 5Q–T, Y, Z). However, the effects are more severe than those resulting from overexpression of Gro^WT^. No *hkb* or *tll* expression is observed at the embryonic termini (Figure 5Q, R, Y). *sna* expression retracts from both termini in these embryos to a greater degree than is observed in embryos overexpressing Gro^WT^ (Figure 5S, Z).

Consistent with the notion that Gro^ΔCR^ has a dominant negative function, overexpression of this variant results in expansion of the expression domains. Slight expansion of *hkb* at the posterior pole of the embryo (Figure 5U, Y) and ventral expansion of *tll* at the anterior pole (Figure 5V, Y) are observed. In the case of *sna*, we observe loss of repression at the poles as well as an increase in the width of the ventral stripe (Figure 5W, Z).

**Figure 6:**
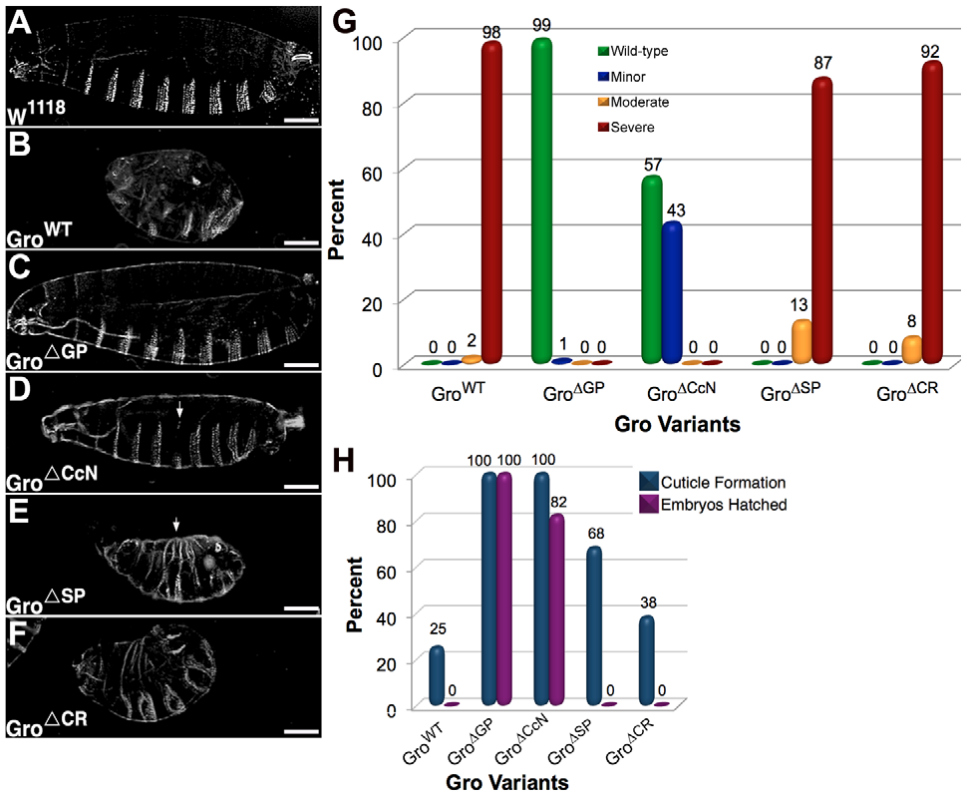
Cuticle phenotypes resulting from overexpression of Gro variants. The *Mat-Gal4* driver was used to drive maternal overexpression of the indicated Gro variants. (A–F) Representative cuticles resulting from overexpression of Gro variants. Cuticles images were obtained using a 10× objective. Scale bars = 100 µm (A) Embryos containing the driver but no UAS-Gro construct show no phenotype. Overexpression of Gro^WT^ (B) and Gro^ΔSP^ (E) resulted in moderate to severe cuticle defects. Overexpression of Gro^ΔGP^ (C) resulted in no defects, while overexpression of Gro^ΔCcN^ (D) resulted in a truncated or missing 4^th^ or 6^th^ abdominal denticle belt (indicted by arrow), a phenotype sometimes observed in weak *gro* hypomorphic embryos, and thus consistent with a weak dominant negative function for this deletion variant. Overexpression of Gro^ΔCR^ (F) often resulted in a pair-rule defect reminiscent of that seen in *eve* mutant embryos. This is consistent with the notion that Gro^ΔCR^ is a dominant negative, since Gro is required for Eve function [35], [61]. (G) Cuticles of 100 embryos laid by females overexpressing each of the five Gro variants were assigned to the following phenotypic categories: Wild-type - no observable defects; Minor defects - 1–2 missing or fused denticle belts; Moderate defects - 3–4 missing and/or fused denticle belts; Severe defects - 5 or more missing denticle belts. (H) 100 embryos overexpressing each variant were scored to determine percent of embryos that deposited cuticle and that hatched.

The cuticle phenotypes resulting from maternal overexpression of the Gro variants are consistent with the effects on target gene expression (Figure 6A–G). While many of the embryos overexpressing Gro^WT^ and Gro^ΔSP^ fail to form cuticle (Figure 6H), those that do show pleiotropic patterning defects, reflecting the repression of multiple genes directing pattern formation (Figure 6B, E). Gro^ΔCR^ overexpression also results in severely defective cuticles, presumably due to a dominant negative function for this variant (Figure 6F). In contrast, overexpression of Gro^ΔGP^ or Gro^ΔCcN^ results in no defects or only mild defects validating the critical roles played by the GP and CcN domains in Gro function (Figure 6C, D).

We conclude that the GP and CcN domains are crucial for Gro-mediated repression in the embryo, while the SP domain serves to temper Gro activity and restrict target gene selection. Furthermore, Gro overexpression modulates target gene expression patterns in addition to target gene expression levels.

### The Gro central region is also required for transcriptional repression in the wing disc

To determine if the above conclusions are applicable in another developmental system, we examined the effect of misexpressing the Gro variants in the developing wing. Anteroposterior (a/p) patterning of the wing imaginal disc requires the Gro-dependent repressor Brk, which silences the expression of genes such as *optomotor blind (omb)* and *vestigial (vg)* at the anterior and posterior edges of the developing wing. As a result, misexpression of Gro in the wing disc results in ectopic repression of a *lacZ* reporter under the control of a *vg* cis-regulatory module termed the *vg* quadrant (*vgQ*) enhancer [1]. To assess the ability of the Gro variants to repress a Brk target, we generated clones of Gro variant overexpressing cells that overlap the *vgQ-lacZ* expression domain.

**Figure 7:**
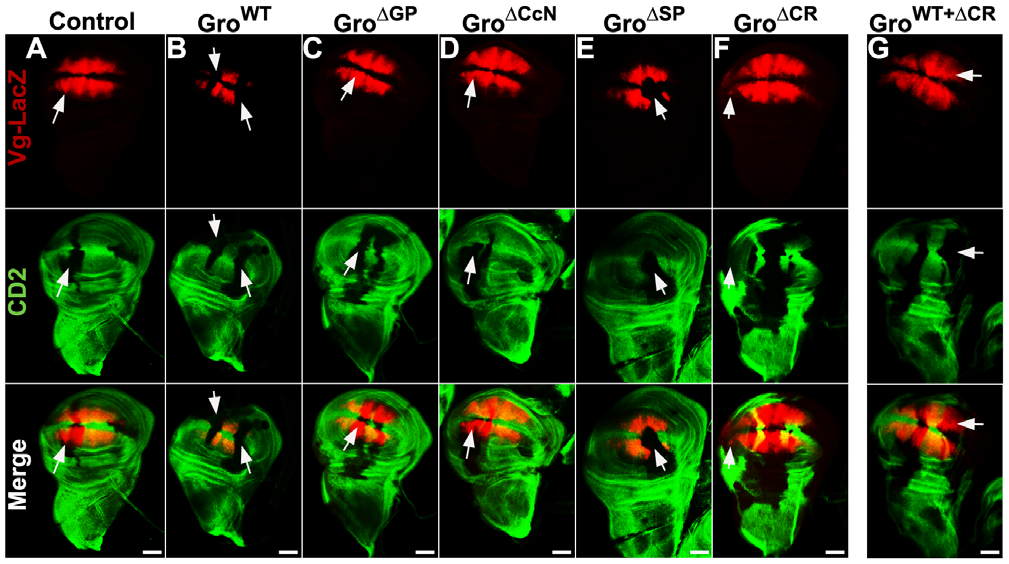
Central domains regulate Gro-mediated repression in the wing disc. Clones of Gro variant overexpressing cells were generated in third instar wing discs contained the *vgQ-lacZ* reporter. Discs were stained with antibodies against CD2 (green) and β-galactosidase (red). Overexpression clones are marked by the absence of CD2. Arrows mark clones that overlap the *vg* expression domain. Gro^WT^ clones (B) and Gro^ΔSP^ clones (E), exhibited ectopic repression of *vgQ-lacZ*, while control clones (A), Gro^ΔGP^ clones (C), and Gro^ΔCcN^ clones (D) exhibited no repression of *vgQ-lacZ*. Gro^ΔCR^ clones (F) exhibited slight expansion of *vgQ-lacZ* into regions in which the reporter is not normally expressed. Clones containing a mixture of overexpressed Gro^WT^ and Gro^ΔCR^ (G) show significantly reduced ectopic repression of *vgQ-lacZ*, relative to clones overexpressing Gro^WT^ alone. Wing disc images were obtained using a 20× objective. Scale bars = 50 µm.

In accord with the results of maternal expression in embryos, clonal misexpression of Gro^WT^ or Gro^ΔSP^ in wing discs results in *vgQ-lacZ* repression (Figure 7B, E, compare to Figure 5A), while misexpression of Gro^ΔGP^ or Gro^ΔCcN^ does not (Figure 7C, D). Misexpression of Gro^ΔCR^ does not lead to repression, but instead leads to a very slight expansion of the *vgQ-lacZ* expression domain into regions at the anterior and posterior edges of the disc where the reporter is not normally expressed (Figure 7F). This is reminiscent of what is observed in *gro* loss-of-function clones [1], [16] suggesting, once again, that Gro^ΔCR^ behaves as a dominant negative form of Gro. This is proven by an experiment in which co-expression of Gro^WT^ and Gro^ΔCR^ significantly attenuates repression by Gro^WT^ (Figure 7G). This dominant negative effect presumably reflects Q domain mediated association of Gro^ΔCR^ with endogenous Gro^WT^.

**Figure 8:**
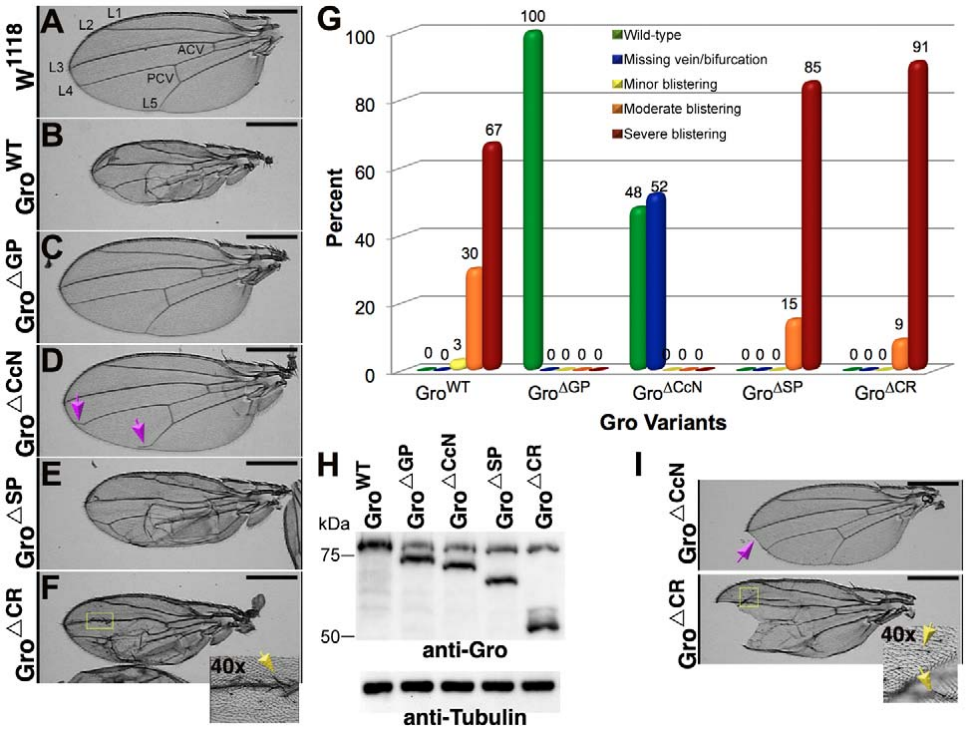
Phenotypes resulting from overexpression of Gro variants in the wing disc. The *Ser-Gal4* driver was used to drive expression of Gro^WT^ or Gro internal deletion variants in the wing. (A) A wing containing the driver but no UAS-Gro construct shows no phenotype. Overexpression of Gro^WT^ (B) and Gro^ΔSP^ (E) resulted in moderate to severe blistering and vein deformation. Overexpression of Gro^ΔGP^ (C) resulted in no defects, while overexpression of Gro^ΔCcN^ (D) resulted in bifurcation of the 4^th^ and/or 5^th^ longitudinal vein (LV, arrows). Overexpression of Gro^ΔCR^ (F) resulted in severe blistering and vein deformation as well as ectopic bristles along the wing vein (inset, yellow arrows). (G) 100 wings of each genotype were scored according to phenotype as indicated showing the differences in the relative severity of the phenotypes generated by overexpression of the various deletion variants. (H) Gro immunoblot of third instar wing discs verifies equal expression levels. Tubulin serves as a control for equal protein levels. (I) In addition to the blistering and wing veination defects, Gro^ΔCcN^ and Gro^ΔCR^ Gro overexpression also resulted in 13% and 29% wing scalloping phenotypes (arrows). The severe defects in the Gro^ΔCR^ overexpressing wings and the milder defects in the Gro^ΔCcN^ overexpressing wings probably results from a dominant negative function for these variant as discussed in the text. The wing scalloping (J) and the ectopic wing bristles (F and I, indicated by arrows in the high magnification insets), both of which are reminiscent of a Notch pathway hypomorphic phenotypes [49]. Except for the high magnification insets in panels F and I, the wing images were obtained using a 4× objective. Scale bars = 400 µm. For the high magnification insets in panels F and I, the images were obtained using a 40× objective.

The adult wing phenotypes resulting from overexpression of the Gro deletion variants in the wing disc are consistent with the conclusions drawn from the *vgQ-lacZ* reporter assays. Figures 8A–F show representative wings from flies of each genotype, while Figure 8G displays the quantitative results of examining multiple discs from flies of each genotype and assigning them to phenotypic classes. All variants were expressed at very similar levels (Figure 8H). In accord with previous studies, we find that expression of wild-type Gro using the *Ser-Gal4* driver (which directs expression in the dorsal compartment of the wing pouch) leads to wing blistering due to inappropriate repression of Gro targets (Figure 8B, G). The GP and CcN domains are required for this overexpression phenotype (Figure 8C, D, G), while the SP domain partially ameliorates it (Figure 8E, G). Finally, overexpression of Gro^ΔCR^ (and to a lesser extent Gro^ΔCcN^) results in wing scalloping and supernumerary wing bristles, which are wing phenotypes reminiscent of those that result from reductions in Notch signaling (Figure 8I) [49]. This is consistent with dominant negative functions for these variants since Gro is required for the function of E(spl) bHLH proteins, which are downstream effectors of Notch signaling [2], [50].

In summary, the wing disc misexpression studies suggest that the positive roles of the GP and CcN domains and the negative role of the SP domain are maintained throughout development.

### A requirement for the GP domain in nuclear localization

**Figure 9:**
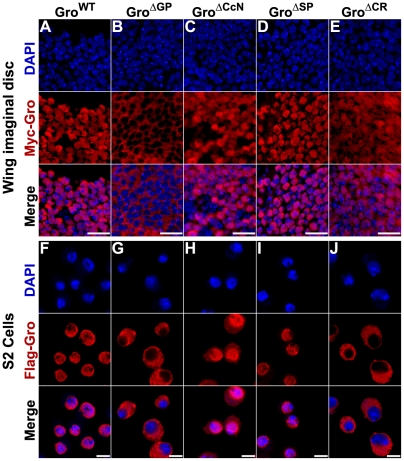
Subcellular localization of Gro central domain deletion variants. (A–E) Third instar imaginal wing discs were stained with Myc antibodies (red) to detect Myc-tagged Gro and DAPI (blue) to stain DNA. Gro^WT^ (A) and Gro^ΔSP^ (D) localized exclusively to the nucleus. Gro^ΔGP^ (B) localized exclusively to the cytoplasm. Gro^ΔCcN^ (C) localized primarily to the nucleus, but we also detected a low level of cytoplasmic localization and Gro^ΔCR^ (E) localized to the nucleus and cytoplasm. Wing disc images were obtained using a 100× objective. Scale bars = 10 µm. (F–J) *Drosophila* S2 cells were stained with FLAG antibodies (red) to detect FLAG-tagged Gro and DAPI (blue) to stain DNA. Gro^WT^ (F) and Gro^ΔSP^ (I) localized exclusively to the nucleus. Gro^ΔGP^ (G) and Gro^ΔCR^ (J) localized exclusively to the cytoplasm. Gro^ΔCcN^ (H) localized primarily to the nucleus. S2 cell images were obtained using a 100× objective. Scale bars = 5 µm.

The CcN domain contains a conserved sequence resembling an NLS, and was thus assumed to be responsible for Gro nuclear import [14]. To determine the requirements of the CcN domain and other domains in nuclear localization, we examined the subcellular localization of central region deletion variants through immunofluorescence imaging of third instar larval wing discs and *Drosophila* S2 cells expressing tagged forms of these variants. Gro^WT^ and Gro^ΔSP^ are exclusively nuclear in both wing discs and S2 cells (Figure 9A, D, F, I). Surprisingly, Gro^ΔGP^ was completely cytoplasmic, while Gro^ΔCcN^ was primarily nuclear, implying that the GP rather than the CcN domain is primarily responsible for nuclear localization (Figure 9B, C, G, H). Therefore, the lack of an overexpression phenotype observed with the Gro^ΔGP^ variant likely reflects, in large part, a failure to localize to the nucleus. These observations also explain why Gro^ΔGP^ does not function as a dominant negative, while Gro^ΔCcN^ does.

Interestingly enough, the Gro^ΔCR^ variant localized to the nucleus and cytoplasm in third instar wing discs (Figure 9E). This is consistent with its strong dominant negative function, but seems to be at odds with the notion that the GP domain is required for nuclear import since Gro^ΔCR^ lacks the GP domain. A plausible interpretation of this finding is that the central region contains a nuclear import signal or interacts with a protein that aids in Gro import in the GP domain and a nuclear export signal somewhere outside this region and that the subcellular localization of Gro depends on the interplay between these two signals. In S2 cells, Gro^ΔCR^ is cytoplasmic suggesting that the relative importance of the import and export signals may be cell type specific (Figure 9J).

### Promiscuous repression by Gro^ΔSP^ could reflect binding to histones

**Figure 10:**
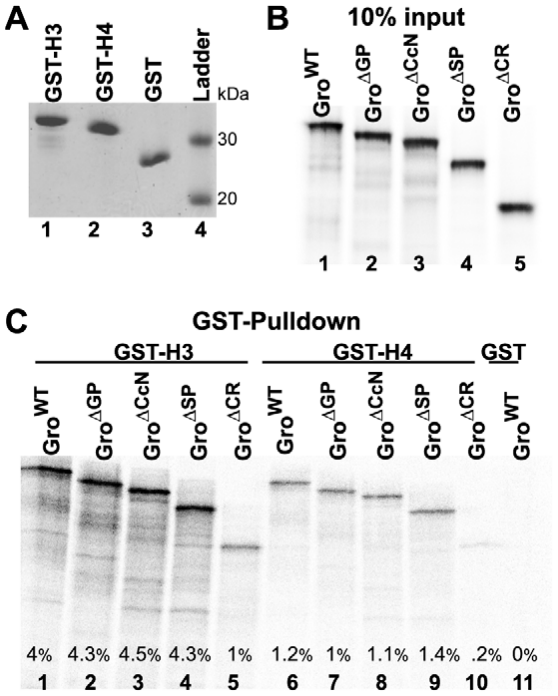
Central region contains redundant determinants of binding to the histone H3 and H4 tails. (A) GST-tagged histone H3 and H4 tails were expressed in bacteria and purified on glutathione beads. 750 ng of GST-H3 (lane 1), GST-H4 (lane 2), and GST (lane 3) were subjected to 10% SDS-PAGE followed by staining with Coomassie Blue. Lane 4 shows size markers with molecular mass indicated in kDa. (B) Gro^WT^ (lane 1), Gro^ΔGP^ (lane 2), Gro^ΔCcN^ (lane 3), Gro^ΔSP^ (lane 4), and Gro^ΔCR^ (lane 5) were translated in vitro in the presence of [^35^S] methionine. 10% of the translation products were subjected to 8% SDS-PAGE and the gel imaged by autoradiography. (C) The translation products from (B) were incubated with glutathione bead bound GST-H3 (Lanes 1–5), GST-H4 (Lanes 6–10), or GST (Lane 11). After extensive washing, bound proteins were eluted with SDS gel sample buffer and subjected to 8% SDS-PAGE. The gel was imaged by autoradiography. The percentage of input protein that bound the immobilized GST fusion proteins is indicated at the bottom of each lane.

The studies presented above suggest that in the absence of the SP domain, Gro can act promiscuously to repress genes that it normally does not repress. This raises the question of how Gro is recruited to such genes. Coimmobilization studies have shown that Gro binds directly to the N-terminal tails of histones H3 and H4 with a preference for the hypoacetylated forms of these histone tails [22], [51]. This histone binding suggests a mechanism for promiscuous recruitment. To explore this possibility further, we examined the ability of the internal deletion variants to bind the H3 and H4 tails. These experiments involved the use of GST-H3 tail and GST-H4 tail fusions (Figure 10A) together with in vitro transcribed and translated Gro deletion variants (Figure 10B).

GST-pulldown assays show that deletion of any single domain was insufficient to inhibit H3 or H4 binding, while deletion of the entire central region reduced H3 and H4 tail binding by at least 4-fold (Figure 10C). Thus, there appear to be multiple redundant determinants of histone binding in the central region, and the observation that Gro^ΔSP^ maintains an ability to bind to histone tails could provide a partial explanation for its promiscuous function.

## Discussion

The findings presented in this paper suggest that the poorly conserved central domains of Gro may play essential roles in the developmental regulation of transcription. Specifically, we have shown that the GP, CcN, and SP domains have important roles in repression that are either essential for viability or greatly increase viability. In these studies, we employed deletion variants that remove entire domains as opposed to point mutations because a previous study suggested that the central region was resistant to point mutagenesis [16]. As discussed further below, we believe that this resistance to point mutagenesis is a reflection of the intrinsic disorder of these domains. Now that we know that the internal domains are essential for function, it will be worthwhile to carry out studies to further dissect the functions of these domains by, for example, generating finer deletions or clustered substitution mutations to map regions responsible for specific protein∶protein interactions.

While the use of deletion variants is subject to the caveat that deletions alter spacing and juxtapose parts of proteins that are not normally juxtaposed, we have demonstrated that our internal Gro deletions do not alter the functions of the adjacent conserved domains, i.e., the self-association function mediated by the Q domain or the repressor binding function mediated by the WD-repeat domain. Furthermore, if the deletions were leading to the misfolding of the conserved domains, we might expect to detect changes in protein stability in vivo, which is something that we do not observe. In addition, the observation that deletion of the CcN domain or of the entire central region leads to dominant negative phenotypes consistent with the known roles of Gro in development (e.g., its role in Notch signaling) suggests that these variants are able to associate normally with endogenous Gro in vivo via a properly folded Q domain. Finally, our finding that deletion of the GP, CcN, and SP domain deletions each result in different phenotypes (see below) is inconsistent with the idea that the only role of these domains is to maintain the spacing between the Q and WD-repeat domains.

### The sensitivity of development to Gro levels

Many of the repressors that interact with Gro (e.g., Dorsal, Cic, and Brk) are distributed in concentration gradients and repress target genes in a concentration dependent manner to direct the formation of multiple distinct domains of gene expression [1], [3], [5], [44]. It is often assumed that Gro merely needs to be present in an active form to allow these repressors to function. Contrary to this idea, however, we find that development is exquisitely sensitive to Gro activity levels. Our overexpression experiments show that a modest increase in Gro levels leads to changes in the spatial domains of target gene expression demonstrating that the threshold concentrations at which targets genes respond to graded repressors are sensitive to Gro activity levels. This is further shown by the sensitivity of development to the *gro* gene dosage as we have shown that flies bearing, one, two, or three copies of the *gro* gene are viable, while a fourth copy results in a loss of viability.

How can we account for the sensitivity of transcription factor gradient threshold responses to Gro activity levels? One possibility is that the assembly of a transcriptionally silent domain is a highly cooperative process that involves the spreading of Gro across a large chromosomal region. A cooperative process of this type might well be highly sensitive to the concentration of active Gro.

### An unanticipated role for the SP domain in target gene specificity

The SP domain apparently functions to keep Gro activity in check as deletion of this domain results in increased repression of Gro targets, decreased target gene specificity (and therefore promiscuous repression), and severely weakened rescue activity. The role of the SP domain in specificity is completely unanticipated since Gro is not a DNA binding protein and therefore would not be expected to have a role in the selection of target genes. However, Gro is capable of binding to chromatin nonspecifically through interactions with histones [22], [34]. This could lead to promiscuous repression. Indeed, we find that Gro^ΔSP^ represses normal Gro targets as well as genes not normally targeted by Gro such as *cin* and *Rpt3*, and therefore a normal function of the SP domain may be to safeguard against promiscuous repression. It could do so, for example, by limiting the ability of Gro to spread along chromatin. If phosphorylation of the SP domain further limits spreading, this would explain how this modification could attenuate repression [28], [30]. Regardless of the mechanism by which the SP domain modulates specificity, our results suggest that co-regulators are likely to be broadly important in target gene selection and cannot be ignored in attempts to predict the network of transcription factor/cis-regulatory module interactions that control development.

### Positive roles for the GP and CcN domains in Gro-mediated repression

Both gain and loss-of-function experiments demonstrate that the GP and CcN domains are essential for Gro function in vivo. Previous studies have suggested that the GP domain is required for the binding of Gro to the histone deacetylase Rpd3 and that Rpd3 function is required for Gro-mediated repression [23], [25], [35]. We thus considered this interaction to be a prime candidate for the essential function of the GP domain. We were surprised to discover, however, that the GP domain has an essential function in nuclear localization. Since the GP domain does not have homology to any known NLS, this suggests the existence of a novel mechanism for Gro nuclear import or that this region interacts with an unknown protein containing a more conventional nuclear localization signal. Because the deletion mutant lacking the GP domain does not enter the nucleus, we are, at this point unable to address the question of whether the GP domain/Rpd3 interaction is required for function in vivo.

Our findings show that although the CcN domain contains a sequence with similarity to a canonical NLS, this domain is not required for nuclear import. This is consistent with previous studies in S2 cells showing that deletion of the putative nuclear localization signal in the CcN domain did not lead to relocalization of the bulk of the Gro to the cytoplasm, whereas deletion of the entire central region did [52]. While the CcN domain is not required for nuclear import, it nonetheless has a critical, but currently unidentified, function in repression.

### Intrinsic disorder in the central domains may enable them to serve diverse regulatory functions

Previous studies identifying lethal alleles of Gro failed to turn up mutations that mapped to the central domains [16]. Despite this fact, the findings presented here demonstrate that the central region plays critical roles in Gro function and in development. It is perhaps not surprising that it is difficult or impossible to identify missense mutations in the Gro central region given the lack of sequence conservation in this region between *Drosophila* Gro and its vertebrate orthologs. This lack of conservation suggests that these domains might not be highly ordered and therefore might be resistant to inactivation by single amino acid changes, a suggestion that is supported by our analysis of the Gro amino acid sequence.

A large fraction of proteins, especially in higher eukaryotes, are thought to contain disordered domains subject to rapid evolution. Many proteins with profound significance to human disease, such as the tumor suppressor p53, contain such domains [39]. Contrary to what is sometimes assumed, this is not a sign that these domains are without required function. Rather the disorder in these domains may play a useful role by allowing them to adopt multiple structural states, which could each mediate interactions with different partners, allowing the domains to function as hubs of large regulatory networks [39]. Thus, by facilitating a large number of regulatory interactions, the disordered central domains in Gro could account for its ability to repress multiple targets via diverse mechanisms. Furthermore, disordered domains may serve as regulatory targets because they can bind their partners with both high specificity and low affinity, a type of binding that can be easily reversed by posttranslational modification [39]. This is consistent with the previous findings showing that the central domains often serve as posttranslational regulatory targets [26], [27], [28], [30], [53].

While the central region in Gro family proteins undergo rapid sequence evolution, their functions are often conserved even when the sequence conservation is hard to recognize. For example, the GP domain appears to be responsible for Rpd3/HDAC1 binding both in *Drosophila* Gro and its mammalian orthologs [23], [24]. Similarly, the SP domain seems to be a target for regulation by some of the same protein kinases in vertebrates and invertebrates alike [28], [30], [31], [53], [54]. Thus, it seems that disordered domains in related proteins may be conserved at the level of function long after evolutionary drift has erased easily recognizable sequence conservation.

### Evidence for target gene-specific mechanisms of Gro-mediated repression

Previous studies suggest that Gro may repress transcription by multiple mechanisms including histone deacetylase-dependent and histone deacetylase-independent mechanisms (see introduction). In accord with this idea, some of the findings presented here suggest that the mechanism of Gro-mediated repression may be target gene specific. For example, we show that *hkb* and *tll* are more sensitive to increases in Gro^WT^ levels than *ftz*. In contrast, the effect of deleting the SP domain is much more dramatic for *ftz* than it is for *hkb* and *tll*. This may reflect gene specific differences in the mechanism of repression that are intrinsic to the structure of the regulatory region of the gene.

In conclusion, it seems that the Gro central domains, although not well conserved, have essential roles in Gro-mediated repression and in the regulation of development. A full understanding of the mechanisms of repression by Gro will require the identification and characterization of the many proteins that are likely to interact with the Gro central region and a determination of the roles of these partner proteins in repression. At least some of these partners may well be target gene specific.

## Materials and Methods

### Plasmid Construction

The plasmids encoding the Gro central domain deletion variants were generated using pET17b-Gro as a template for PCR [17]. The PCR primers used to generate the ΔGP, ΔCcN, ΔSP, and ΔCR deletions are listed in Table S1. The resulting PCR products contained the entire pET17b vector and sequences encoding all of Gro with the exception of the domain being deleted, and contained an AscI restriction site at the site of the deletion.

To generate transgenic UAS constructs that can be expressed in somatic and germ line cells, PCR fragments encoding Gro^WT^, Gro^ΔGP^, Gro^ΔCcN^, Gro^ΔSP^, and Gro^ΔCR^ and that also contained 5′ NotI and 3′BamHI restriction sites were inserted into the pUASP transformation vector [55]. PCR products encoding the above Gro variants that contained 5′ XhoI and 3′ StuI restriction sites were inserted into the p131 pUAST transformation vector so that the Gro coding region was in frame with the amino-terminal 6XMyc epitope tag [56].

To generate S2 cell expression constructs, PCR fragments encoding Gro^WT^, Gro^ΔGP^, Gro^ΔCcN^, Gro^ΔSP^, and Gro^ΔCR^ with an N-terminal FLAG tag (DYKDDDDK) and containing 5′ XhoI and 3′ SpeI restriction sites were inserted into the S2 cell expression acceptor vector pMK33-BD [57].

A Gro genomic rescue construct bearing a 10,000 bp *gro*-containing fragment from the right arm of chromosome 3 (spanning genomic coordinates 21,866,400 to 21,876,400) was created using the attP-attB-P[acman] recombineering system [42]. The left and right homology arms were amplified with primers sets 1 and 2 (Table S1) using BAC13F13 (Chori) as the DNA template and then inserted into the transformation vector attB-P(acman). SW102 cells containing BAC13F13 were then transformed with linearized attB-P(acman) containing the homology arms. Accurate gap repair was verified with primer sets 3 and 4 (Table S1) and the correctly recombined plasmid (named attB-P(acman)-*gro* 10 kb).

To generate genomic rescue constructs encoding the Gro central domain deletions Gro^ΔGP^, Gro^ΔCcN^, Gro^ΔSP^, we used attB-P(acman)-*gro* 10 kb as a PCR template. Primers used to generate these rescue constructs are given in Table S1. PCR amplification products encoding the genomic regions to the left and right of the site of the deletions were inserted into attB-P(acman). All rescue constructs were introduced into flies containing an attP docking site located at 2L-22A by phiC31 site-specific transgenesis (Rainbow Transgenic Flies, Inc., fly stock 9752 (22A)).

### Disorder prediction algorithms

Disorder probability in Gro was predicted using the PONDR-FIT™ and FoldIndex© algorithms [40], [41]. These algorithms take advantage of the discovery that intrinsically disordered proteins have significantly different amino acid sequences than do ordered proteins. Specifically, disordered proteins display low sequence complexity, a low content of bulky hydrophobic amino acids, and a high proportion of charged and polar amino acids. The algorithms were developed using databases of known disordered and ordered proteins to train artificial neural networks to assign protein disorder scores to moving windows of amino acids across proteins [39]. For the PONDR-FIT™ algorithm, regions displaying scores consistently less than 0.5 are likely to be ordered, while regions displaying scores consistently greater than 0.5 are likely to be disordered. For the FoldIndex© algorithm, regions displaying scores consistently below 0 are likely to be disordered, while regions displaying scores consistently above 0 are likely to be ordered.

### Gro genomic rescue experiments

The progeny of flies containing one of two lethal *gro* mutant alleles, *gro^MB12^* (a strong hypomorph) or *gro^MB36^* (a null) [16], balanced over *TM3* and a rescue construct balanced over *CyO* were examined to determine the fraction that were homozygous for the *gro* mutant allele. The expected ratio for 100% rescue is one-third *gro*/*gro* to two-thirds *gro*/*TM3* progeny.

### Embryo and wing disc immunoblots

To examine Gro expression in embryo, 50 embryos produced by the female progeny of a pUASP-Gro×*Mat-Gal4* cross were placed in 40 ul of SDS-PAGE sample buffer (60 mM Tris-Cl [pH 6.8], 2% SDS, 10% glycerol, 5% β-mercaptoethanol, and 0.01% bromophenol blue), mashed, and boiled. Samples were analyzed by 8% SDS-PAGE and probed with either a 1∶500 dilution of mouse anti-Gro monoclonal antibody (Hybridoma Bank) or a 1∶10,000 dilution of mouse anti-tubulin monoclonal antibody (Sigma) using the Millipore dry blot method. Blots were subsequently incubated with a 1∶10,000 dilution of secondary antibody conjugated to horseradish peroxidase (CalBiochem) and signal was detected by enhanced chemiluminescence (ECL) with SuperSignal West Pico substrates (Pierce). To examine Gro expression in wing discs, forty third instar wing imaginal discs from the progeny of a pUASP-Gro×*Ser-Gal4* cross were placed into 30 ul of SDS-PAGE sample buffer. Samples were processed and analyzed as described for the embryo immunoblots.

### Coimmobilization assays with His-tagged Gro variants

pET17b-Gro [17] was used to express wildtype full-length Gro, and pET17b-Gro^ΔGP^, pET17b-Gro^ΔCcN^, pET17b-Gro^ΔSP^, pET17b-Gro^ΔCR^ were used to express the Gro central domain deletion variants. pET3c-His-tagged N-terminal Gro (2–194) wild-type, Gro (2–194) 40/89, and Gro (2–194) 38/87 [18] were used to express wild-type and point mutants of GroN fused to a 6xHis-tag. For cotranslation, constructs encoding two forms of Gro were mixed before being added to the TNT T7 quick-coupled transcription-translation system (Promega) in the presence of [^35^S]-methionine. 10% of the translation product was reserved for analysis of the input, while the remaining 90% was diluted into binding buffer (25 mM HEPES [pH 7.6], 450 mM NaCl, 10 mM imidazole, 0.1% Tween 20, 1 mM dithiothreitol) and incubated with Nickel-Nitrilotriacetate (Ni-NTA) beads (QIAGEN) overnight at 4°C. Beads were then washed extensively with binding buffer. Proteins bound to the beads were eluted with SDS-PAGE sample buffer. Samples and reserved input material were analyzed by 10% sodium dodecyl sulfate-polyacrylamide gel electrophoresis (SDS-PAGE) and autoradiography.

### GST-pulldown assays

GST-histone tail fusion proteins were expressed in bacteria and purified as described previously using plasmids pGEX-2T-H3, pGEX-2T-H4, and pGEX-2T-control [58]. Gro deletion variants were translated in vitro from the pET17b constructs in the presence of [^35^S]-methionine using the TNT T7 coupled reticulocyte lysate system (Promega). In vitro translated Gro variants were incubated overnight with GST fusion proteins immobilized on glutathione beads at 4°C in HEMNK buffer (40 mM HEPES at pH 7.5, 5 mM MgCl2, 0.2 mM EDTA, 1 mM DTT, 0.5% NP-40, 0.1 M KCl). Following binding, the beads were washed extensively with HEMNK buffer. Proteins were eluted in SDS-PAGE sample buffer and the eluates, as well as the 10% reserved input material, were resolved by 8% SDS-PAGE and visualized by autoradiography. GST-Brk fusion proteins were expressed with pGEX-5x-1-Brk(441–589) [18] and pGEX-5xl-control vectors as described for the GST-histone tail fusions, except that the cells were lysed by two passes through a microfluiditor in STE buffer (0.15 M NaCl, 10 mM Tris-Cl, pH 8, 1 mM EDTA) with 5 mM DTT, 1% Triton-X 100 and protease inhibitors. Lysed cells were cleared by centrifugation. GST-pulldown assays to examine binding to vitro translated Gro deletion variants were performed as described for the GST-histone tail pulldown experiments, except the binding buffer was PBS containing 340 mM NaCl, 1 mM EDTA, 0.1% NP-40, and 10% BSA and the washes were with PBS containing 290 mM NaCl. Proteins were eluted in SDS-PAGE sample buffer and the eluate and 2% reserved input material were resolved by 8% SDS-PAGE and visualized by autoradiography.

### Preparation of embryonic cuticles

*Mat-Gal4* virgin females were crossed with transgenic males containing UASp constructs encoding Gro deletion variants or with control *w^1118^* males and incubated at 25°C. Embryos were collected from F1 females for 3 hours and placed at 25°C for 24 hours to allow completion of embryonic development and cuticle deposition. Cuticles were prepared as described previously [59]. Briefly, embryos were washed, devitellinized in1∶1 heptane∶methanol, and placed on a slide. 3∶1 lactic acid∶water was added to the embryos and the slide incubated at 60°C for 24 hours. Cuticles were imaged on a Zeiss Axioscope microscope in darkfield with a 10× objective.

### Preparation of adult wings

*Ser-Gal4* virgin females were crossed with transgenic males containing UAS constructs encoding Gro deletion variants or with control *w^1118^* males and incubated at 25°C. Shortly after eclosion, adult wings from the F1 generation were dissected from the flies, washed in methanol, and mounted in 70% glycerol. Adult wings were imaged on a Zeiss Axioscope microscope in brightfield with a 4× objective.

### Immunofluorescence

For subcellular localization of Gro variants, S2 cells stably transformed with the pMK33 vectors encoding the FLAG tagged Gro variants and induced with 0.5 mM CuSO_4_ were stained with 1∶250 diluted mouse anti-FLAG antibodies (Sigma), and wing discs expressing Myc-tagged Gro variants were stained with 1∶400 diluted mouse anti-Myc antibodies (Santa Cruz Biotechnology). Secondary antibodies were goat anti-mouse conjugated with Alexa Fluor 568 (Molecular Probes). DNA was stained with 1 ug/ml DAPI. Confocal images of S2 cells and imaginal discs were obtained on a TCS SPE confocal laser-scanning microscope (Leica Microsystems, Heidelberg) using the 100× and 20× objectives, respectively.

### Fluorescence in situ Hybridization

*Mat-Gal4* virgin females were crossed with transgenic males containing UAS constructs encoding Gro deletion variants or with control *w^1118^* males and incubated at 25°C. Embryos containing maternally overexpressed Gro deletion variants were subjected to multiplex fluorescence in situ hybridization (FISH) as described previously [60] using a mixture of digoxigenin-11-UTP (DIG) labeled *hkb* antisense RNA probe, Biotin-16-UTP (BIO) labeled *tll* antisense RNA probe, and Fluorescein-12-UTP (FITC) labeled *sna* antisense RNA probes (Roche). Primary antibodies were 1∶300 diluted sheep anti-DIG and 1∶500 diluted mouse anti-FITC. Secondary antibodies were 1∶400 diluted donkey anti-sheep conjugated to Alexa Fluor 555, 1∶400 diluted rabbit anti-mouse conjugated to Alexa Fluor 633, and 1∶400 diluted anti-BIO conjugated to Alexa Fluor 488 (Molecular Probes). Embryos were mounted with ProLong Gold antifade reagent and DAPI (Invitrogen) and images were obtained on a TCS SPE confocal laser-scanning microscope (Leica Microsystems, Heidelberg) using a 20× objective.

### Quantitative Reverse-Transcriptase Polymerase Chain Reaction (qRT-PCR)

RNA was isolated from 0–3 hour embryos containing maternally overexpressed Gro deletion variants using Trizol reagent (Invitrogen), subjected to RQ1 DNase treatment (Promega), and repurified by repeating the Trizol and phenol-chloroform extractions followed by isopropanol precipitation. 3 ug of RNA was used to make cDNA with an oligo(dT)_12–18_ primer (Invitrogen), M-MLV Reverse Transcriptase (Invitrogen), and RNaseOUT (Invitrogen). cDNA was then diluted 10-fold and added to a 3.6 uM mixture of primer pairs and 2× FastStart SYBR Green mix (Roche). cDNA levels were quantified by qPCR using an Opticon Monitor 3 system (Bio-Rad) and normalized to RpL32 [25]. Fold repression was then determined by dividing these values into the RpL32 normalized levels from control RNA made from embryos lacking maternally overexpressed Gro variants. Statistical significance of each value relative to the value in embryos lacking overexpressed Gro was determined from the two-tailed unpaired Student's T-test. Primers used for *gro*, *zen*, *twi*, *dpp*, *ftz*, *hkb*, *tll*, *sna*, *kni*, *Rpt3*, and *cin* are given in Table S2.

## Supporting Information

Table S1 **Primers used to generate Gro constructs.**
(DOC)

Table S2 **Primers used for qRT-PCR.**
(DOC)
