# Supplementary material for: The Unconserved Groucho Central Region Is Essential for Viability and Modulates Target Gene Specificity
Source: PLoS One. 2012 Feb 3;7(2):e30610. doi: 10.1371/journal.pone.0030610 (PMC3272004; doi:10.1371/journal.pone.0030610)
Supplement: Table S1 — Primers used to generate Gro constructs. (DOC) [file pone.0030610.s001.doc]

***Supporting Information Table***

**Table S1. Primers used to generate Gro constructs.**

| **Primer Name** | **Primer sequence*** |
| --- | --- |
|  | **Primers for creating pET17b vectors encoding Gro deletion variants** |
| **GP 3'-5'** | atatt*ggcgcgcc*CTGCTGGGCGTGGATCTGTTGCCCA |
| **GP 5'-3'** | atatt*ggcgcgcc*gGTTTCGCCGGCCGATCGTGAGAAGT |
| **CcN 3'-5'** | atatt*ggcgcgcc*CGAATTGCGCAATCGCTCCTCGGC |
| **CcN 5'-3'** | atatt*ggcgcgcc*gGAGGTGCGCGATCGGGAAAGCTTGA |
| **SP 3'-5'** | atatt*ggcgcgcc*CATAGACACGTGCTCGCCGTTGGGA |
| **SP 5'-3'** | atatt*ggcgcgcc*gGGAAAGCCTGCATACTCTTTCCATATGAACGGC |
|  |  |
|  | **Primers for Gro attB-P(acman) rescue construct recombineering **** |
|  | **Primer set 1** |
| **LA-AscI-*gro*-F** | attac*ggcgcgcc*GTCTACAAGAACTTGCAGCAATTCCAC |
| **LA-BamHI-*gro-*R** | AAACCGCCCAGCGACATGG*ggatcc*CCAAAATAGATTTCCATTGTCAGAGCA |
|  | **Primer set 2** |
| **RA-BamHI-*gro*-F** | TGCTCTGACAATGGAAATCTATTTTGG*ggatcc*CCATGTCGCTGGGCGGTTT |
| **RA-PacI-*gro*-R** | accac*ttaattaa*GGAGCTGCAATATTTTGCCAGCC |
|  | **Primer set 3** |
| **LA-seq-*gro*-F** | CAATTTACTTTATTGGGATGTTGGG |
| **RA-seq-*gro*-R** | TCAGACACTTCCTGCAGCTCAC |
|  | **Primer set 4** |
| **5’-check-*gro*-R** | GTGAACCGTTAAGGACATTACCG |
| **3’-check-*gro*-F** | GCTGAAAATGGCATTCTTGAAGA |
|  |  |
|  | **Primers for generating deletion variant rescue constructs** |
| ***gro*-F-left-5'** | attat*gcggccgc*GTCTACAAGAACTTGCAGCAATTCCAC |
| ***gro*-R-right-3'** | attat*gcgatcgc*GGAGCTGCAATATTTTGCCAGCC |
| **GP-R-left-3'** | CGAAAC*ggatcc*CTGCTGGGCGTGGATCTGTTG |
| **GP-F-right-5'** | CAGCAG*ggatcc*GTTTCGCCGGCCGATCGT |
| **CcN-R-left-3'** | CACCTC*ggatcc*CGAATTGCGCTGCAAGATCAG |
| **CcN-F-right-5'** | AATTCG*ggatcc*GAGGTGCGCGATCGGGAAA |
| **SP-R-left-3'** | CTTTCC*ggatcc*CATAGACACGTGCTCGCCGT |
| **SP-F-right-5'** | TCTATG*ggatcc*GGAAAGCCGTAAGTCATTTATGTCAAT |

* Upper case letters indicate sequences corresponding to portions of the *gro* sequence, while lower case letters represent additional sequences added to introduce restriction sites (italicized)

** Primer sets 1 and 2 were used for amplifying the left and right homology arms, respectively. Primer set 3 checks for correct triple ligation between the homology arms and attB-P(acman) vector. Primer set 4 was used to confirm correct retrieval of the *gro* genomic region by gap repair.
