# Supplementary material for: The Unconserved Groucho Central Region Is Essential for Viability and Modulates Target Gene Specificity
Source: PLoS One. 2012 Feb 3;7(2):e30610. doi: 10.1371/journal.pone.0030610 (PMC3272004; doi:10.1371/journal.pone.0030610)
Supplement: Table S2 — Primers used for qRT-PCR. (DOC) [file pone.0030610.s002.doc]

***Supporting Information Table***

Table S2. Primers used for qRT-PCR.

| **Primer Name** | **Primer Sequence** |
| --- | --- |
| ***zen 5'*** | AAGTCGGCTCCTATTCAGCA |
| ***zen 3'*** | TTGTAGTTGGGAGGCAGACC |
| ***twi 5'*** | TTCAAGTCCCTGCAGCAGAT |
| ***twi 3'*** | CGGCACAGGAAGTCAATGTA |
| ***dpp 5'*** | AGCTCGACTCGGTCAACATC |
| ***dpp 3'*** | CGGGAATGCTCTTCACGTC |
| ***ftz 5'*** | AGCCAGGAGATCAATCATCG |
| ***ftz 3'*** | CTGGTAGCGGGTGTACGTCT |
| ***hkb 5'*** | AAGTTCAAGTGCCCCAACTG |
| ***hkb 3'*** | CATGTGTTCACGTCGCACTT |
| ***tll 5'*** | CACATCGGTATGCAGTCGTC |
| ***tll 3'*** | TGCAGGGCACATGGTATAGA |
| ***sna 5'*** | TGCACCATCACTATTGCGTAG |
| ***sna 3'*** | CACCAAAACCGAATCGACTTA |
| ***kni 5'*** | TGAACCAGACATGCAAAGTG |
| ***kni 3'*** | GCTGATGGTGCTGATGTTGT |
| ***Rpt3 5'*** | ACGAACTGGACATGGAGGAT |
| ***Rpt3 3'*** | CTTTTTCAGGTTTCGCTGCT |
| ***cin 5'*** | AGTGCTCCCTTTCTGTGCTT |
| ***cin 3'*** | ACGCTCGCGTATAACTCTCC |
| ***gro 5'*** | AAGTTCACCATCGCCGATAC |
| ***gro 3'*** | GCATCTCCGTCTTCTCGTTC |
